# Targeting protein-protein interactions for therapeutic discovery via FRET-based high-throughput screening in living cells

**DOI:** 10.1038/s41598-018-29685-z

**Published:** 2018-08-22

**Authors:** Daniel R. Stroik, Samantha L. Yuen, Kevyn A. Janicek, Tory M. Schaaf, Ji Li, Delaine K. Ceholski, Roger J. Hajjar, Razvan L. Cornea, David D. Thomas

**Affiliations:** 1 Department of Biochemistry, Molecular Biology, and Biophysics, University of Minnesota, Minneapolis, Minnesota 55455 USA; 2 Cardiovascular Research Center, Icahn School of Medicine at Mount Sinai, New York City, New York, 10029 USA

## Abstract

We have developed a structure-based high-throughput screening (HTS) method, using time-resolved fluorescence resonance energy transfer (TR-FRET) that is sensitive to protein-protein interactions in living cells. The membrane protein complex between the cardiac sarcoplasmic reticulum Ca-ATPase (SERCA2a) and phospholamban (PLB), its Ca-dependent regulator, is a validated therapeutic target for reversing cardiac contractile dysfunction caused by aberrant calcium handling. However, efforts to develop compounds with SERCA2a-PLB specificity have yet to yield an effective drug. We co-expressed GFP-SERCA2a (donor) in the endoplasmic reticulum membrane of HEK293 cells with RFP-PLB (acceptor), and measured FRET using a fluorescence lifetime microplate reader. We screened a small-molecule library and identified 21 compounds (Hits) that changed FRET by >3SD. 10 of these Hits reproducibly alter SERCA2a-PLB structure and function. One compound increases SERCA2a calcium affinity in cardiac membranes but not in skeletal, suggesting that the compound is acting specifically on the SERCA2a-PLB complex, as needed for a drug to mitigate deficient calcium transport in heart failure. The excellent assay quality and correlation between structural and functional assays validate this method for large-scale HTS campaigns. This approach offers a powerful pathway to drug discovery for a wide range of protein-protein interaction targets that were previously considered “undruggable”.

## Introduction

A major goal of drug discovery in recent years is the development of small molecules that target specific protein-protein interactions^1^, but there is a growing consensus that such targets are intrinsically difficult to perturb specifically with small molecules^2^. In the present study, we focus on the interaction of phospholamban (PLB), a 52-residue single-pass transmembrane protein expressed in the sarcoplasmic reticulum (SR) of cardiac muscle, and its regulatory target SERCA2a, the cardiac SR Ca-ATPase. SERCA2a is responsible for removing Ca from the cytosol into the SR, inducing muscle relaxation^3^. In its enzymatic cycle, the Ca-ATPase undergoes a transition from a high Ca affinity (E1) to a low Ca affinity (E2) conformation, with ATP binding and autophosphorylation powering the calcium transport process. PLB is in dynamic equilibrium between homopentamers and monomers, where the oligomeric state is proposed to act as a reservoir^4^. Monomeric PLB reduces the apparent Ca affinity of SERCA2a, and this inhibitory effect is relieved by β-adrenergic stimulation of PLB phosphorylation, thus providing a Ca-transport reserve to enhance cardiac performance. In heart failure (HF), however, there is a Ca-transport deficit that leads to elevated sarcoplasmic Ca (cytotoxic) and incomplete relaxation and filling of the ventricle (diastolic dysfunction), as well as incomplete SR re-filling with Ca, which blunts Ca release (systolic dysfunction)^5^. The market is saturated with preload and afterload reducers that provide symptomatic relief, but there is an urgent need for an effective and safe cardiotonic therapy that directly targets deteriorated diastolic and systolic function. Since HF depends on multiple factors and causes, numerous strategies have been developed to mitigate or reverse cardiac dysfunction. A promising approach is to enhance cardiac muscle contractility by modulating Ca transport^6,7^. The SERCA2a-PLB interaction is widely viewed as an attractive target for cardiovascular therapeutic discovery and development, to correct the pathophysiological myocyte state and its consequences to cardiac function.

It is well established that decreased SERCA2a activity, as seen in HF animal models and human patients, results in slower and less complete muscle relaxation after each contraction^8–11^. Recent efforts using gene therapy to increase SERCA2a activity accomplish this either through SERCA2a overexpression or by reducing SERCA2a inhibition by PLB^12,13^. SERCA2a activation is tolerated in healthy animal models and significantly enhances cardiac function in numerous models of heart disease^14,15^. These results validate SERCA2a activation for HF therapy. SERCA2a overexpression via recombinant adeno-associated virus (rAAV) was achieved in patients experiencing end-stage HF in a recent phase II clinical trial^16^. Despite encouraging preliminary results^17^, the trial failed to meet its primary end goals, due to dosage constraints and fundamental limitations of rAAV gene therapy; *e.g*., in patients with pre-existence of neutralizing antibodies^18^.

We have pursued an alternative approach to activate SERCA2a using small-molecule drugs that decrease SERCA2a inhibition by PLB. This small-molecule approach is designed to overcome limitations associated with gene therapy^18^ and is amenable to acute, non-invasive hospital intervention with the potential for chronic usage to improve cardiac contractility. As PLB is almost exclusively expressed in the heart^4^, compounds that specifically target the SERCA2a-PLB complex will be inherently tissue-specific, thus reducing the risk of adverse side effects. Some previous attempts to discover compounds that activate SERCA have been largely unsuccessful, due to reliance on low-throughput, low-precision assays of ATPase activity^19^. We previously reported a fluorescence resonance energy transfer (FRET)-based high-throughput screening (HTS) method using purified SERCA and PLB labeled with fluorescent dyes in a reconstituted membrane system^20^. A 20,000-compound screen was performed on this *in vitro* sample using steady-state (intensity) fluorescence detection and identified the first SERCA activators. Surprisingly, none of these compounds directly affected the SERCA2a-PLB interaction^20^. We hypothesize that detection of compounds that disrupt the SERCA2a-PLB requires a more precise detection technique.

**Figure 1:**
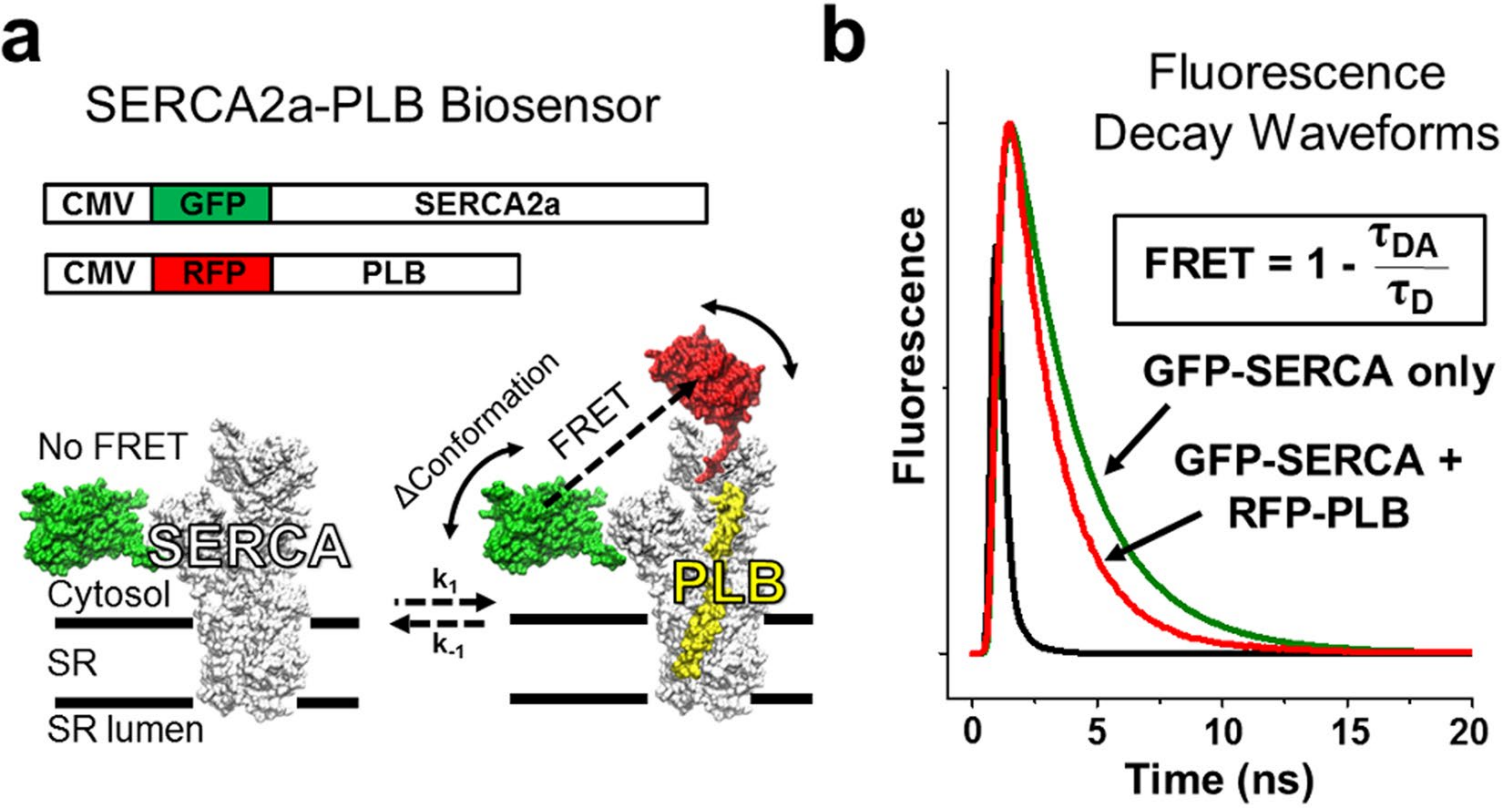
Structure-based high-throughput screening to target SERCA2a-PLB complex. (**a)** Schematic diagram illustrating expression vectors and structural model of GFP-SERCA2a free or bound to RFP-PLB. **(b)** Time-resolved fluorescence waveforms as a readout to measure FRET between GFP-SERCA2a and RFP-PLB. FRET is calculated as the change between lifetimes (exponential decay times) of donor-only and donor-acceptor samples (τ_D_ and τ_DA_).

Here, we introduce an HTS platform that directly monitors the SERCA2a-PLB interaction using time-resolved FRET (TR-FRET) between GFP-tagged SERCA2a and RFP-tagged PLB constructs expressed in a transformed human cell line (Fig. 1a). When RFP-PLB (acceptor) is bound to GFP-SERCA2a (donor), FRET is detected as a decrease in donor fluorescence lifetime (FLT), which is calculated from fluorescence decay waveforms (Fig. 1b). The FRET measurement is a direct readout of changes in PLB binding to SERCA2a and/or the structure of the SERCA2a-PLB complex. The R^−6^ distance dependence of FRET makes it sensitive to protein-protein interactions and subtle structural changes. We applied this structure-based SERCA2a-PLB biosensor to drug discovery, by performing a triplicate screen of the Library of Pharmacologically Active Compounds (LOPAC, 1280 compounds), using an FLT plate reader provided by Fluorescence Innovations, Inc^21,22^. The FLT measurement yields a 30-fold decrease in the coefficient of variation (CV = standard deviation/mean) compared with intensity detection used in our previous screen^20^, resulting in an HTS platform of excellent quality. To assess the effectiveness of this HTS platform, we performed FRET concentration-response and ATPase activity assays, revealing ten Hit compounds that reproducibly affect SERCA2a-PLB structure and function. This strong correlation between structural and functional assays validates this method to discover SERCA2a-PLB effectors for large-scale HTS campaigns.

## Results

### Expression, localization, and FRET readout of SERCA2a-PLB biosensor

**Figure 2:**
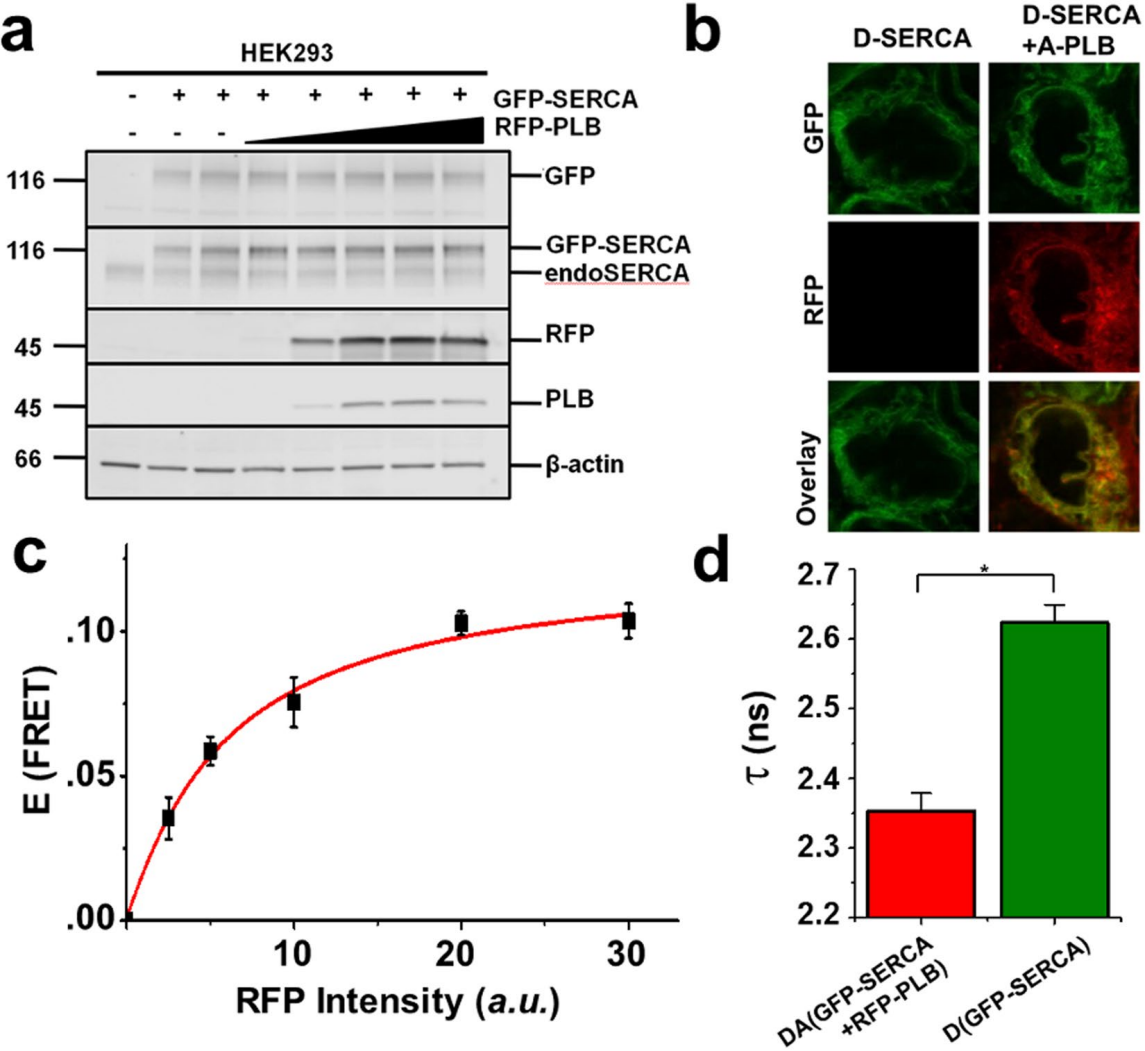
Biochemical and spectroscopic characterization of SERCA2a-PLB biosensor. (**a**) Immunoblots of homogenates from untransfected HEK293 cells (lane 1), cells expressing GFP-SERCA2a (lanes 2-3), or cells expressing GFP-SERCA2a and increasing amounts of RFP-PLB (lanes 4–8). Antibodies from top to bottom are anti-GFP, anti-SERCA2, anti-RFP, anti-PLB and anti-β-actin, and the image has been cropped. (**b**) Confocal fluorescence imaging of HEK293 cells expressing GFP-SERCA2a (left) or GFP-SERCA2a and RFP-PLB (right). (**c**) FRET efficiency E from GFP-SERCA2a (donor) to RFP-PLB (acceptor) shows hyperbolic dependence on acceptor concentration. (**d**) The mean FLT in the presence of saturating acceptor is τ_DA_ = 2.35 ± 0.02 ns, corresponding to E = 0.103 ± 0.004. Error bars indicate SD (n = 3). Statistical differences determined using the Student’s t test: *P ≤ 0.01.

To monitor the SERCA2a-PLB interaction in a natural membrane environment, we developed a biosensor system by fusing GFP and RFP to the N-termini of SERCA2a and PLB, respectively, and measured FRET changes as a readout of changes in the SERCA2a-PLB complex structure and binding. Attaching fluorescent proteins (FP) at these sites does not interfere with the activity of SERCA or PLB^23^. HEK293 cells were transfected with GFP-SERCA2a and RFP-PLB constructs, and expression was verified by SDS-PAGE and immunoblot (Fig. 2a). The GFP-tagged SERCA protein was resolved from untagged SERCA, as verified by a GFP-specific antibody. There were no other bands of lower mobility, and there was no aggregation apparent in intact cells. To specifically detect the SERCA2a-PLB interaction, a monomeric mutant of PLB^24^ was used, with three mutations (C36A, C41F, and C46A) in the transmembrane domain. The structure and function of this PLB variant is indistinguishable from those of monomeric wild-type PLB^25,26^. Transfection of this RFP-PLB variant produced a single band recognized by PLB- and RFP-specific antibodies. The SERCA2a-PLB biosensor showed the expected endoplasmic reticulum localization by confocal microscopy (Fig. 2b) with no bright puncta (which would have indicated aggregation) or other non-uniformities. FRET between GFP-SERCA2a and RFP-PLB in live cells showed hyperbolic dependence on protein concentration, with a maximum energy transfer efficiency E (fractional decrease of FLT or intensity) of 0.103 ± 0.004 (Fig. 2c,d). The observed nanosecond time-resolved fluorescence decay was best fitted by a two-Gaussian distance distribution, centered at R_1_ = 5.6 ± 1.6 nm and R_2_ = 9.8 ± 1.9 nm (Figure S1)^27–29^. The measured distances are in agreement with previous TR-FRET measurements^30^.

**Figure 3:**
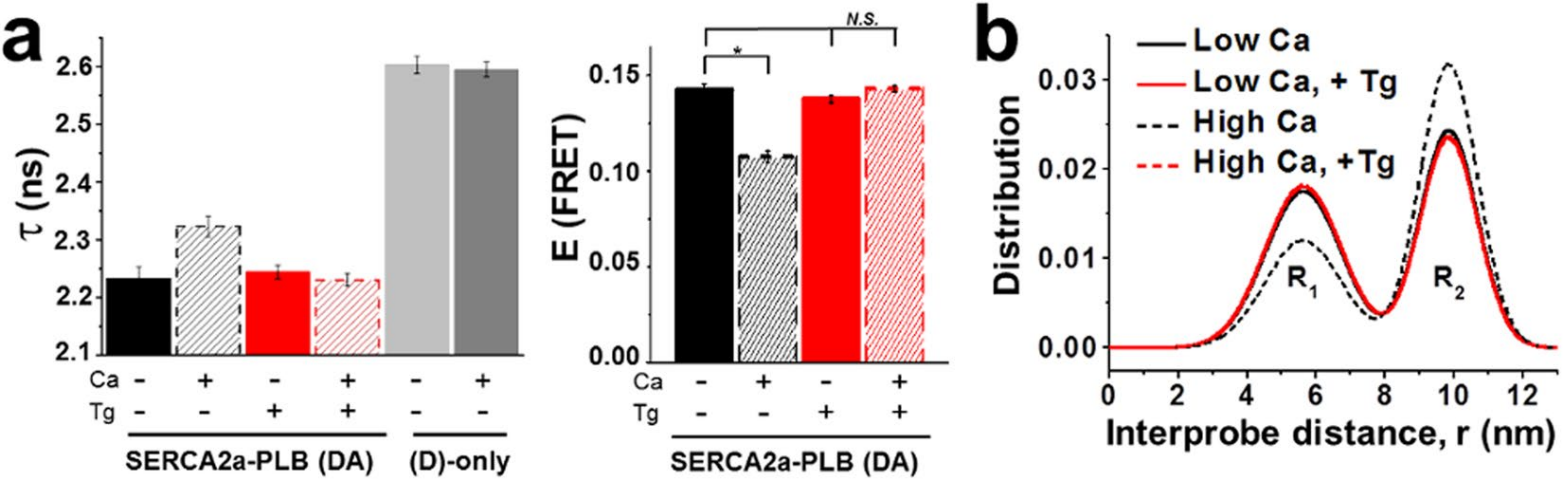
Effects of Ca and Tg on SERCA2a-PLB biosensor. (**a**) Left- FLT measurements in saponin-permeabilized cells under conditions of low Ca (100 nM) and high Ca (10 µM), and in the absence and presence (100 nM) of the SERCA inhibitor thapsigargin (Tg). Right- FRET readout calculated from (**a**) shows a Ca-dependent decrease in FRET, that is ablated by addition of Tg. Error bars indicate SD (n = 3). Statistical differences determined using 1-way ANOVA followed by Tukey post hoc analysis (more detailed analysis is provided in Supplemental Table 4). *P ≤ 0.01 and *N.S*. indicating P ≥ 0.05. (**b**) Distance distributions between donor on SERCA and acceptor on PLB, based on Eq. (10).

PLB inhibition of SERCA is relieved by elevated Ca^4^. The most likely explanation, supported by *in vitro* measurements of FRET between SERCA and PLB, is that relief of inhibition occurs primarily through a structural rearrangement of the bound complex and not through disassociation^23,31,32^. Specifically, SERCA-PLB FRET is reduced in the presence of a high Ca concentration but is not ablated^23,31^. To test this, we quantified FRET changes in permeabilized cells under conditions of low Ca (100 nM) and high Ca (10 µM). In agreement with previous studies, high Ca resulted in a modest FRET decrease (from E = 0.14 to E = 0.11), confirming that the binding interface is altered by structural changes but that the SERCA2a-PLB complex remains intact (Fig. 3a). Treatment with thapsigargin (Tg), a SERCA inhibitor that shifts the transition between the E1/E2 conformations toward E2, prevents the Ca-dependent decreases in FRET (Fig. 3a). We consistently observe a decreased DA lifetime in saponin-treated cells and cell homogenates relative to the live cell assay (Fig. 2d); this may reflect changes in membrane dynamics or the fluorophore environment^33^. As both Tg and low Ca promote the E2 conformation, we hypothesized that we would be able to resolve the distance distribution associated with this conformational state by TR-FRET. Indeed, we see a shift in the equilibrium of the structural states from the short distance (R_1_) to the long distance (R_2_) under high-Ca conditions, and this is ablated by Tg (Fig. 3b). This demonstrates that the SERCA2a-PLB biosensor is sensitive to changes in the protein complex interactions, and thus suitable for our HTS platform.

### HTS of LOPAC library to identify compounds that affect SERCA2a-PLB FRET

**Figure 4:**
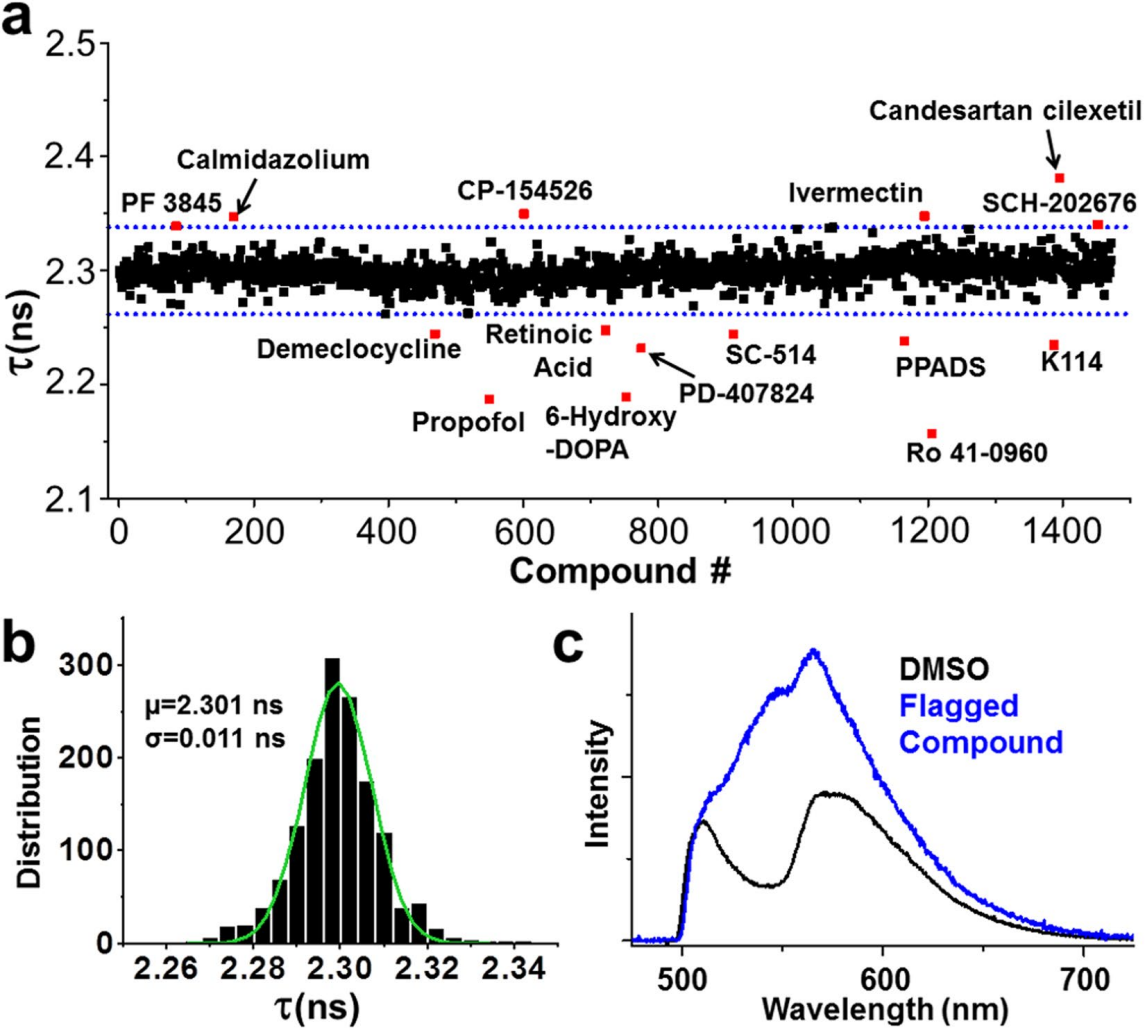
High-throughput screen results. Compounds were screened in triplicate at a final concentration of 10 µM. (**a**) FLT values from one representative screen including DMSO control wells with a Hit threshold (>3 S.D. of mean) indicated by dotted blue lines. (**b**) Gaussian fit of the FLT distribution. (**c**) SERCA2a-PLB biosensor visible emission spectrum upon excitation at 473 nm (black). Addition of a fluorescent compound greatly alters the spectrum (blue).

To assess the performance of our FRET-based HTS method, we screened a library of 1280 small-molecule compounds (LOPAC) for identification of SERCA2a-PLB modulators. This library is suitable for a pilot screen, as it covers all major drug target classes and most compounds are commercially available, satisfactory for the requirements of orthogonal screening^34^. Assay quality was determined based on controls (DMSO-only samples) on each plate, as indexed by the Z’ factor from Eq. (2)^21,35^. The high precision enabled using FLT measurements provides an excellent HTS assay quality (Z′ = 0.80 ± 0.01)^21^. We incubated test compounds with live cells expressing the SERCA2a-PLB biosensor in a 384-well assay format^36^, and selected Hits that changed FLT (>3S.D.) of the GFP donor fused to SERCA2a in triplicate screens of the library (red points in Fig. 4a). The distribution of FLT for the DMSO-control wells is normal and centers at 2.30 ns (Fig. 4b). Because FLT measurements are susceptible to interference from fluorescence of the test compound itself, we also acquired fluorescence emission spectra by scanning the same 384-well plates using a recently developed spectral plate reader^32^ (Fig. 4c) and applied a spectral similarity index to flag fluorescent compounds^36,37^. In total, 21 compounds were identified as Hits, which altered FRET from SERCA2a to PLB in at least 2 replicates after filtering out compounds with interfering fluorescence (false positives). This equates to a ~1.6% Hit rate, which is in the acceptable range between 0.5–3% to maintain a manageable number for post-HTS testing through orthogonal assays^38^.

### FRET concentration-response assays

**Figure 5:**
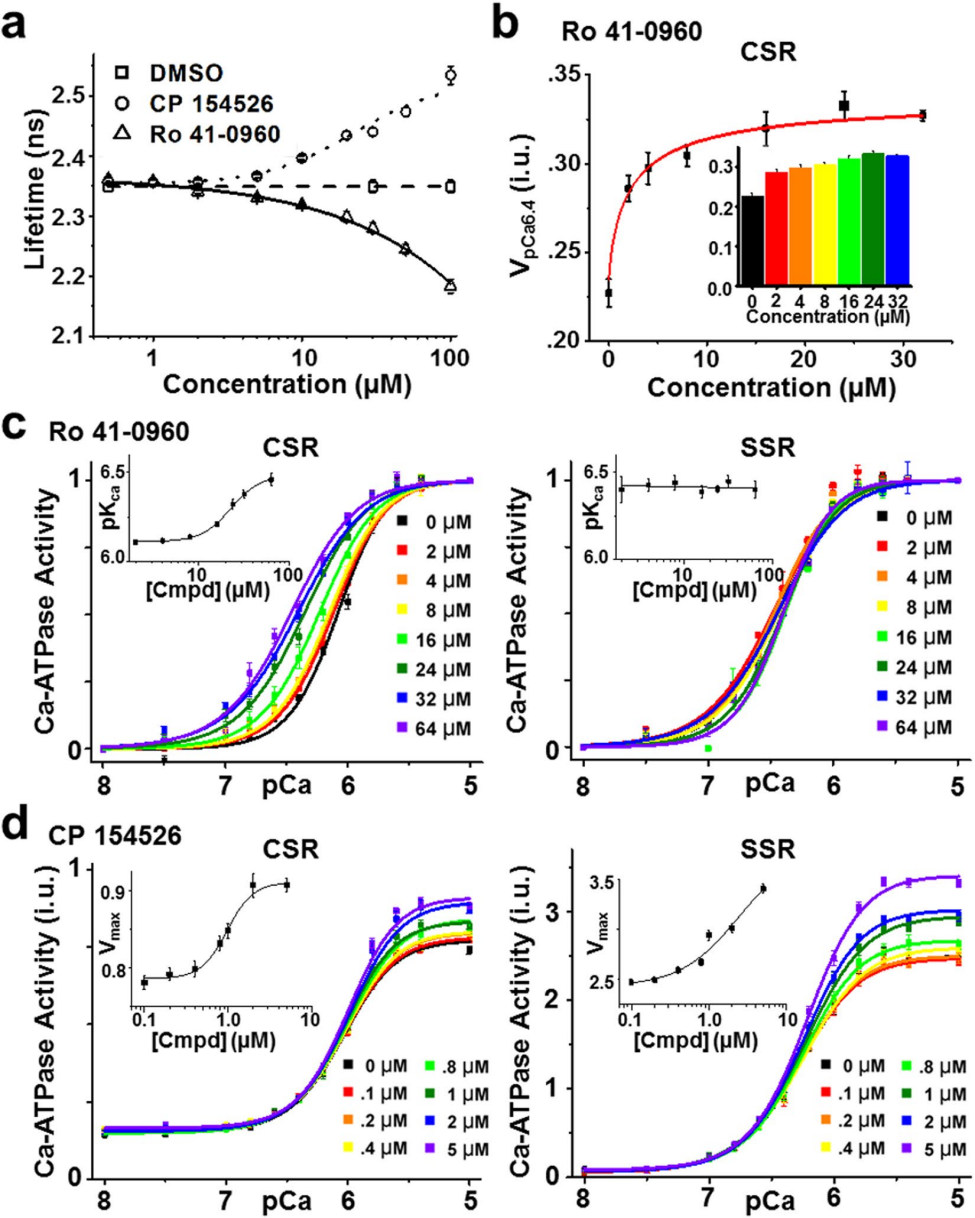
Effects of SERCA activators identified in this screen. (**a**) FRET dependence on (0.01–100 µM) CP 154526 (circle) and Ro 41-0960 (up-triangle) under conditions similar to those in the primary HTS. (**b**) (inset) Dependence of CSR Ca-ATPase activity at pCa 6.4 on the concentration of Ro 41-0960. Error bars indicate SD (n = 3). Statistical differences determined using 1-way ANOVA followed by Tukey post hoc analysis (more detailed analysis is provided in Supplemental Table 5). (**c**) Normalized Ca-ATPase activity of cardiac SR (CSR) and skeletal SR (SSR) was measured after a 20 min incubation in the presence of Ro 41-0960 (up to 64 µM) or DMSO control. Error bars indicate SEM (n = 3). (inset) Concentration response of SERCA apparent Ca affinity (pK_Ca_). (**d**) Ca-ATPase activity of CSR and SSR after 20 min incubation in the presence of CP 154526 (up to 5 µM) or DMSO control. Error bars indicate SEM (n = 3). (inset) Concentration response for maximum velocity (V at pCa 5; V_max_).

The Hit compounds were further tested in concentration-response (0.01–100 µM compound) FRET assays under the same conditions as used in the primary screens, and 17 compounds displayed concentration-dependent changes in FRET (Table S1). Of these, 5 compounds (tenidap, FPL 64176, CP 135807, olprinone, and X80) did not reach saturation of FRET, as evidenced by high EC_50_ values (>100 µM) when fit to Eq. (13). It is possible that these compounds are not intrinsically fluorescent but are affecting the GFP FLT by changing the fluorophore environment or structure. The remaining 12 compounds had EC_50_ values below 100 µM (Fig. 5a, Figure S3, Table S1) and cause either an increase in FRET (*e.g*., Ro 41-0960) or decrease in FRET (*e.g*., CP 154526, ivermectin, SCH-202676) of the SERCA2a-PLB biosensor. Thus, the FRET dose-response assay can function as an effective tool to refine the list of Hits and limit the number of compounds to be tested via medium-throughput activity assays in >50 K compound screens.

### Functional effects of structural (FRET) Hits on Ca-ATPase function

To characterize the relationship between the structural readout (FRET) and the functional effects, *in vitro* Ca-ATPase assays were performed on cardiac SR (which contains both SERCA2a and PLB). During diastole and systole, the intracellular Ca concentration oscillates between 0.1 and 1 µM (pCa 7 to pCa 6)^4^. PLB does not alter basal or maximal SERCA2a activity but inhibits ATPase activity within this range of Ca concentration. An initial functional screen to identify compounds that disrupt PLB inhibition was carried out by measuring Ca-ATPase activity at an intermediate Ca concentration (pCa = 6.4). Ro 41-0960 increased activity by 44% (at 24 µM) in cardiac SR in this Ca-ATPase assay (Fig. 5b), while 8 compounds were inhibitors under these conditions.

To further examine the functional effects of Hit compounds, Ca-ATPase assays were performed on both skeletal SR (SERCA1a only) and cardiac SR (SERCA2a + PLB) at a series of Ca concentrations ranging from pCa 5 to 8. We expect two classes of activators to be useful for the treatment of HF: (1) a compound that increases SERCA’s apparent Ca affinity (pK_Ca_) due to relief of PLB inhibition, which would display a leftward shift in the V vs. pCa plot in cardiac SR only and (2) a compound that increases V_max_ (SERCA activity at saturating Ca). We tested all 21 Hits identified in the HTS-assay for Ca-ATPase activity and found 10 Hits that reproducibly affect SERCA activity (2 activators and 8 inhibitors). The activators included Ro 41-0960 and CP-154526 (chemical structures in Figure S2). Ro 41-0960 increased Ca affinity (pK_Ca_) in cardiac SR (SERCA2a + PLB) from 6.05 to 6.45 in a concentration-dependent manner (EC_50_ = 22.98 µM), but had no effect on Ca affinity in skeletal SR (SERCA1a only), even at the highest compound concentrations (Fig. 5c), indicating that the functional effects are specific to the SERCA2a-PLB complex. Notably, SERCA’s Ca affinity at saturating [Ro 41-0960] in cardiac SR is equivalent to the Ca affinity in skeletal SR, suggesting that the compound fully reverses PLB inhibition. The second activator discovered, CP-154526, increased V_max_ by 34% in cardiac SR (EC_50_ = 1.0 µM) and 35% in skeletal SR (EC_50_ = 2.5 µM) (Fig. 5d).

**Figure 6:**
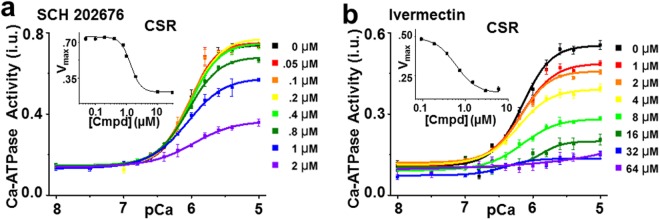
Ca-ATPase activity for SERCA inhibitors. (**a**) ATPase activity from CSR) was measured after 20 min incubation with SCH 202676 (up to 2 µM) or DMSO control. (inset) Concentration response for limiting activity at high Ca (V_max_). (**b**) ATPase activity from CSR after 20 min incubation in the presence of ivermectin (up to 64 µM) or DMSO control. (inset) Concentration response for limiting activity at high Ca (V_max_).

In addition to the SERCA activators, 8 inhibitor compounds were found in our screen. Based on the design of the assay to detect the SERCA-PLB interaction, we expected that the functional effects of Hit compounds would be to increase the apparent Ca affinity (similar to Ro 41-0960). Surprisingly, the majority (80%) of Hit compounds that had functional effects were found to inhibit SERCA. The most likely explanation is that these compounds bind to the SERCA2a-PLB complex and induce inhibitory alterations in the structure of the complex, consistent with changes in FRET between fluorophores attached to SERCA2a and PLB. These compounds inhibit SERCA activity in both skeletal (SSR, SERCA1a in the absence of PLB) and cardiac SR (CSR, SERCA2a in the presence of PLB). From the FRET measurements alone, we cannot determine potential binding interfaces of the compound to SERCA, but we speculate that these compounds do not bind to the SERCA2a-PLB interface, but rather affect FRET through allosteric changes in SERCA itself. Also, we cannot rule out the possibility of isoform-dependent activities (SERCA1a vs SERCA2a); although recent crystal structures (PDB ID = 5MPM) demonstrate strong similarity between the isoforms. Figure 6 highlights the results from the two most effective SERCA inhibitors (chemical structures in Figure S2). SCH 202676 decreased V_max_ by 68% (EC_50_ = 1.4 µM) in cardiac SR and 97% in skeletal SR (EC_50_ = 0.9 µM) (Fig. 6a, Figure S4). The antiparasitic ivermectin decreased V_max_ by 78% (EC_50_ = 6.2 µM) in cardiac SR and 88% in skeletal SR (EC_50_ = 5.8 µM) (Figure b, Figure S4). These results agree with a previous report showing inhibition of skeletal muscle SR Ca-ATPase by ivermectin^39^, with 90% of the activity being inhibited at high concentrations (50 µM). Overall, there is strong correlation between structural (FRET-based) and functional (Ca-ATPase-based) results. Moreover, we found a diversity of chemotypes (Figure S2) and functional outcomes of the tested Hit compounds.

## Discussion

The Ca re-uptake process is deficient in cardiomyocytes in both experimental and human heart failure^40–44^. Therapies targeting the underlying molecular causes have the potential to significantly restore heart function^6,7^. SERCA2a sequesters Ca into the SR of cardiomyocytes, and its function is regulated through protein-protein interactions with PLB^3^. A decrease in expression of SERCA2a is a hallmark of heart failure, and gene therapy approaches to restore its levels back to normal have been successful in experimental models but not so far in clinical trials^13,16,17,45–47^. The relevance of the SERCA2a-PLB complex in human heart function is underscored by the existence of patients who develop cardiomyopathies due to mutations in the *PLN* (PLB-encoding) gene^48–50^. Some of the PLN mutations have opposite sub-cellular mechanisms of action. One of them, R9C, results in chronic inhibition of SERCA2a by mutant PLB and early death, which is consistent with the known mechanisms on PLB regulation and the findings in mice^48,51^. The other mutation is associated with loss of PLB function (L39stop) and results in dilated cardiomyopathy and premature death in the homozygous, opposite to what is seen in mice^50^. The interaction between SERCA2a and PLB is complex and may be species dependent. Despite great importance, the development of SERCA2a-PLB specific therapies has been limited, largely due to the lack of sensitive and rapid methods for screening compound libraries that disrupt or alter the protein-protein interface.

The present report addresses this problem through the development of a structure-based HTS method using the GFP-RFP FRET pair fused to the human isoforms of SERCA2a and PLB, expressed in live cells. Of the 10 Hits that reproducibly altered SERCA2a-PLB structure and function in this report, most of the compounds (9) have not been previously identified to affect Ca-ATPase activity. The one exception is ivermectin, a broad-spectrum antiparasitic compound. Although it is non-toxic to animals at applied doses, it has been reported that ivermectin, along with other macrocyclic lactones, inhibits SERCA activity at higher doses^39^. Ivermectin appears to inhibit all SERCA isoforms (1a^39^, 2a^52^, and 2b^39^), as well other mammalian P-type ATPases (Na^+^, K^+^ and H^+^/K^+^). Our results support these findings, as we see inhibition of skeletal SR (containing SERCA1a) and cardiac SR (containing SERCA2a and PLB) with EC_50_ values of 5.8 µM and 6.2 µM, respectively. Most importantly, two of the Hit compounds identified are SERCA activators. These include Ro 41-0960, which increases the apparent Ca affinity of SERCA2a, as seen in a leftward shift in the Ca-ATPase assay data, and CP 154526, which increases the enzyme’s maximal activity at high [Ca]. This confirms that this live-cell HTS platform can detect compounds that act to alter the SERCA2a-PLB interaction and also compounds that act on SERCA independently of PLB.

SERCA1a is also subject to regulation by sarcolipin (SLN) in skeletal muscle. SLN functions by decreasing Ca affinity of SERCA and partially uncoupling Ca transport from ATP hydrolysis, and is proposed to bind to a similar transmembrane interface of SERCA as PLB (groove formed by M2, M6, and M9 helices) with distinct orientations of the PLB/SLN cytoplasmic domains. Of the compounds tested in this pilot screen, no compound had effects on SSR exclusively that would be expected upon changes in the SERCA1a/SLN interaction, such as increased Ca affinity. Future studies with RFP-labeled SLN may be needed to discover such compounds.

The advancement of fluorescent-protein biosensors as tools for drug discovery has generated new classes of reagents that report specific molecular processes within the intracellular milieu that have been previously inaccessible to HTS^53^. FRET techniques are sensitive to changes in intra- and inter- molecular distances and can thus report relatively small changes in protein structures and protein-protein interactions. As a result, cell-based FRET assays have been developed to monitor a range of biological activities including membrane and cytoplasmic processes^54,55^. The application of FLT measurements and analysis allows the detection of subtle FRET changes that would be missed by intensity-based technology^20,21,56^. Indeed, the majority of our Hit compounds caused small FRET efficiency changes, ranging 0.02 to 0.06. The sensitivity of the assay to compounds of the SERCA2a-PLB functional interaction is indicated by the observation that ~50% of compounds tested that altered SERCA2a-PLB FRET in a concentration-dependent manner also directly affected SERCA function, as measured by an *in vitro* ATPase assay.

The position of the fluorophore tagged to a protein can alter its function and may not report the exact position of certain domains of the tagged protein. This concern is especially relevant for FPs because of their large size. On the other hand, the fact that the fluorophore of GFP and RFP is buried within the barrel allows the fluorophore to maintain a homogenous chemical environment. Attachment of FPs to SERCA and PLB did not alter catalytic activity nor its ability to localize to the membrane^23^, including the GFP-RFP FRET pair used in this study^30^. Still, it is necessary to understand that FRET measurements report the proximity between fluorophore tags and only indirectly on the connected protein domain arrangement. Because it is possible that the position of the GFP and/or RFP molecules are affected by assay conditions or the presence of test compounds, addition controls are needed. It is also possible that compounds may be able to quench the donor fluorescence without emitting detectable fluorescence themselves, as would be detected in the visible spectrum (Fig. 4c). As a reference for this possibility, we have screened the LOPAC library under similar conditions (*e.g*., final [compound] = 10 µM) using HEK cells expressing the GFP-RFP FRET pair connected by a flexible linker or GFP-SERCA2a (donor) alone. Compounds that altered the FLT greater than 3 S.D. in these controls were excluded from further consideration, and not included in the 21 Hit compounds reported here. Lastly, separation distance changes or modulation in dipole-dipole interactions can engender changes in FRET. Because the rotational correlation time of FPs is longer than observed fluorescent decays, this brings into question whether to assume dynamically averaged dipole-dipole orientations (*e.g*., κ^2^ = 2/3) or a static isotropic model^57^. Vogel *et al*., compares the dependence of <E> for CFP on the assumption of dynamic and static models, and demonstrates that the apparent FRET values observed (between 0.10–0.15) for the SERCA2a-PLB biosensor are similar assuming either model^58^. Further, the κ^2^ parameter was calculated by simulation for a SERCA molecule with CFP attached at the N-terminus using a similar length linker as used in this study^59^. The orientation factor is 0.6, which is slightly lower than what is predicted by a dynamic model^59^.

Considering this, the Hit compounds identified cause relatively small changes in the FRET readout and do not eliminate the FRET signal, implying that subtle domain movement of SERCA2a and/or PLB is sufficient to influence enzymatic function. This also suggests that these compound-dependent conformational changes occur while PLB is still bound to SERCA2a. This is consistent with reports detecting at least two distinct PLB conformations exist while bound to SERCA, each with opposing functional outcomes (Subunit Model)^27,31,60–62^.

All these compounds are different from those identified in an earlier screen, where we used a 2-color SERCA construct^21,36,63^. Thus, this SERCA2a-PLB HTS assay could be used in combination with the SERCA-only assays, to enrich the spectrum of SERCA-modulator chemotypes. More generally, the approach illustrated here is applicable to any protein-protein interaction target for which there is sufficient information about structure and function to design FRET biosensors that are sensitive to changes in the protein complex interactions.

## Methods

### Molecular Biology

eGFP and tagRFP were fused to the N-terminus of human SERCA2a and human PLB, respectively, as described previously^21,36,64^. We have previously demonstrated that attachment of the fluorescent proteins at these sites does not interfere with the activity of SERCA^21,63^. Native PLB equilibrates between monomers and homopentamers^65^. To simplify the system and ensure that we are specifically measuring the SERCA2a-PLB interaction, a monomeric mutant of PLB^24^ was used, with three mutations (C36A, C41A, and C46A) in the transmembrane domain. All mutations were introduced by Quikchange mutagenesis (Agilent Technologies, Santa Clara, CA) and sequenced for confirmation.

### Cell Culture

Human embryonic kidney cells 293 (HEK293, ATCC, Manassas, VA) were cultured in phenol red–free Dulbecco’s modified Eagle’s medium (DMEM) (Gibco, Waltham, MA) supplemented with 2 mM l-glutamine (Invitrogen, Waltham, MA), heat-inactivated 10% fetal bovine serum (FBS HI, Gibco). Cell cultures were maintained in an incubator with 5% CO2 (Forma Series II Water Jacket CO2 Incubator, Thermo Fisher Scientific, Waltham, MA) at 37 °C. To generate the one color human SERCA2a stable cell line, HEK293 cells were transiently transfected using Lipofectamine 3000 (Invitrogen). Transiently transfected cells were treated with G418 (Sigma-Aldrich, St. Louis, MO) for two weeks to select for expressing cells. Stable cell lines expressing 2C-SERCA were screened for uniform population by flow cytometry and fluorescence microscopy. To generate SERCA2a-PLB biosensor, HEK293 cells were transiently transfected using Lipofectamine 3000 with GFP-SERCA2a and RFP-PLB in a 1:7 molar ratio for the screen. Cells were then assayed 48 hours post-transfection. For cell permeabilization experiments, cells were trypsinized (TrypLE, Thermo Fischer Scientific), PBS-washed (three times), and resuspended in a homogenization buffer (20 mM MOPS, 0.5 MgCl_2_, protease inhibitor cocktail (Roche, Basel, Switzerland)). Saponin (Sigma-Aldrich) was added to a final concentration of 10 µg/mL and cells were incubated for 4 min at 37 °C water bath. Saponin was removed by centrifugation for 3 min, cells were washed twice with homogenization buffer, and resuspended in homogenization buffer.

### Western Blot

Samples were separated on a 4–20% polyacrylamide gradient gel (Bio-Rad, Hercules, CA) and transferred to polyvinylidene difluoride membrane. The membrane was blocked in Odyssey Blocking Buffer (LI-COR Biosciences, Lincoln, NE) followed by overnight incubation of the primary antibody rabbit anti-GFP (1:1000; ab290, Abcam, Cambridge, United Kingdom)^66^, mouse anti-SERCA2 (1:1000; 2A7-A1, Abcam)^21^, rabbit anti-tagRFP (1:1000; ab233, Evrogen)^67^, mouse anti-PLB (1:1000, 2D12, Abcam)^64^ or rabbit anti-β-actin (1:5000, ab8227, Abcam)^68^ in the cold room. Blots were incubated with anti-mouse or anti-rabbit secondary antibodies conjugated to IRDye 680RD or IRDye 800CW, respectively, for 1 h at room temperature (1:20,000; LI-COR Biosciences). Blots were quantified on the Odyssey scanner (LI-COR Biosciences). Full-length blots are presented in Supplementary Figure S5.

### Compound Handling and Preparation of 384-Well Assay Plates

The LOPAC compounds (Thermo Fisher Scientific) were received in 96-well plates and reformatted into 384-well polypropylene intermediate plates (Greiner Bio-One, Kremsmunster, Austria) using a multichannel liquid handler, BioMek FX (Beckman Coulter, Miami, FL), and then transferred to 384-well Echo Qualified source plates (Labcyte, Inc., Sunnyvale, CA). Assay plates were prepared by transferring 50 nL of the 10 mM compound stocks or DMSO from the source plates to 384-well black polypropylene plates (Greiner), using an Echo 550 acoustic dispenser (Labcyte). LOPAC compounds were formatted in four plates, with the first two and last two columns loaded with 50 nL of DMSO and used for compound-free controls. These assay plates were then sealed and stored at −20 °C prior to usage. Cells were dispensed (50 µL) using a Multidrop Combi liquid dispenser from Thermo (Pittsburg, PA), at a density of 10^6^ cells/mL in PBS.

### Fluorescence Data Acquisition

FLT measurements were conducted in a prototype top-read FLT-PR designed and built by Fluorescence Innovations, which reads each 384-well plate in ~3 min. GFP donor fluorescence was excited with a 473 nm microchip laser from Concepts Research Corporation (Belgium, WI), and emission was acquired with 490 nm long-pass and 520/17 nm band-pass filters (Semrock, Rochester, NY). This instrument uses a unique direct waveform recording technology that enables high-throughput FLT detection at high precision^69^. We have previously demonstrated the performance of this plate reader with known fluorescence standards, as well as with a FRET-based HTS method that targets SERCA^22,36,37^.

### HTS Data Analysis

TR-fluorescence waveforms for each well were fitted based on a two-exponential decay function using least-squares minimization global analysis software^22^. FRET efficiency (E) was determined as the fractional decrease of donor FLT (τ_D_), due to the presence of acceptor fluorophore (τ_DA_):

1 $${E}=1-\frac{{{\tau }}_{{\boldsymbol{DA}}}}{{{\tau }}_{{\boldsymbol{D}}}}$$

Assay quality was determined based on controls (DMSO-only samples) on each plate, as indexed by the Z′ factor^35^:

2 $${Z}^{\prime} =1-\frac{3({{\sigma }}_{{DA}}+{{\sigma }}_{{D}})}{|{{\mu }}_{{DA}}-{{\mu }}_{{D}}|}\,$$

where σ_D_ and σ_DA_ are the standard deviations (SDs) of the controls τ_D_ and τ_DA_, respectively, and μ_D_ and μ_DA_ are the means of the controls τ_D_ and τ_DA_, respectively. A compound was considered a Hit if it changed E by >3 SD relative to that of control samples (E_0_) that were exposed to 0.1% DMSO. The 3 SD Hit selection threshold is typical for normally distributed HTS data, whereby 0.27% of the readings are expected to fall outside this limit. The threshold may be further adjusted to constrain the number of Hits according to the resources available for evaluation via secondary (orthogonal) assays.

### Time-Resolved FRET

TRF waveforms from donor and FRET-labeled samples were analyzed as described in our previous publications^69–71^. The measured time-resolved fluorescence waveform,

3 $${I}({\boldsymbol{t}})={\int }_{-\infty }^{\infty }{IRF}({t}-{t}^{\prime} )\cdot {F}({t}^{\prime} ){dt}^{\prime} $$

is a function of the nanosecond decay time t, and is modeled as the convolution integral of the measured instrument response function, IRF(t), and the fluorescence decay model, *F*(t). The fluorescence decay model

4 $${{F}}_{{D}+{A}}({t})={{x}}_{{D}}{{F}}_{{D}}({t})+(1-{{x}}_{{D}})\,{{F}}_{{DA}}({t})$$

is a linear combination of a donor-only fluorescence decay function *F*_D_(t) and an energy transfer-affected donor fluorescence decay *F*_DA_(t). The donor decay *F*_D_(t) is a sum of *n* exponentials

5 $${{F}}_{{D}}({t})=\sum _{{i}=1}^{{n}}{{A}}_{{i}}\exp (-{t}/{{\tau }}_{{i}})$$

with discrete FLT species τ_i_ and pre-exponential mole fractions *A*_i_. For the GFP donor, two exponentials (*n* = 2) were required to fit the observed fluorescence. The energy transfer-affected donor decay function, *F*_DA_(t),

6 $${{F}}_{{DA}}({t})=\sum _{{j}=1}^{{N}}{{X}}_{{j}}\cdot {{T}}_{{j}}({t})$$

is a sum over multiple structural states (*j*) with mole fractions *X*_j_, represented by FRET-affected donor fluorescence decays *T*_j_(t). The increase in the donor decay rate (inverse donor FLT) due to FRET is given by the Förster equation

7 $${{k}}_{{Ti}}={{k}}_{{Di}}{({R}/{{R}}_{0{i}})}^{-6},$$

where

8 $${k}_{{\rm{DAi}}}={k}_{{\rm{Di}}}+{k}_{{\rm{Ti}}},\,$$

and

9 $${k}_{Di}=1/{\tau }_{i}$$

We modeled TR-FRET assuming that each structural state *j* (Eq. (6) corresponds to a Gaussian distribution of interprobe distances, ρ_j_(*R*):

10 $${T}_{j}(t)={\int }_{-\infty }^{\infty }{\rho }_{j}(R)\cdot \sum _{i=1}^{n}{A}_{i}exp\,(\frac{-t}{{\tau }_{i}}\cdot [1+{(\frac{{R}_{0i}}{R})}^{6}])dR$$

11 $${\rho }_{{\rm{j}}}(R)=\frac{1}{{\sigma }_{{\rm{j}}}\sqrt{2\pi }}\,\exp \,(\frac{-{[R-{R}_{{\rm{j}}}]}^{2}}{2{\sigma }_{{\rm{j}}}^{2}})$$

12 $${\sigma }_{j}=FWH{M}_{j}/(2\sqrt{2\,\mathrm{ln}\,2})$$

### Isolation of Sarcoplasmic Reticulum Vesicles

Skeletal muscle SR membrane vesicles were isolated from longissimus dorsi obtained from New Zealand white rabbits, as previously described^31^. Cardiac SR membrane vesicles were isolated from ventricular myocardium obtained from fresh pig hearts^72^. All experimental protocols were reviewed and approved by University of Minnesota’s Institutional Animal Care and Use Committee, an Association for Assessment and Accreditation of Laboratory Animal Care institution.

### Ca-ATPase Activity

An enzyme-coupled, NADH-linked ATPase assay was used to measure SERCA ATPase activity in 96-well microplates. Each well contained 0.8 μg (skeletal) or 2 μg (cardiac) of SR vesicles adjusted for the different SERCA contents of skeletal and cardiac SR, 50 mM MOPS (pH 7.0), 100 mM KCl, 5 mM MgCl_2_, 1 mM EGTA, 0.2 mM NADH, 1 mM phosphoenol pyruvate, 10 IU/mL of pyruvate kinase, 10 IU/mL of lactate dehydrogenase, 3.5 μg/mL of the Ca ionophore A23187, and CaCl_2_ added to set free [Ca] to the desired values. The assay was started upon the addition of ATP at a final concentration of 5 mM and read in a SpectraMax Plus microplate spectrophotometer rom Molecular Devices (Sunnyvale, CA). The Ca-ATPase assays were conducted over a range of [Ca], and the ATPase activities were fitted using the Hill function

13 $$V=\frac{{V}_{max}}{1+10-(n(p{K}_{ca}-pCa))}$$

where V is the initial ATPase rate, V_max_ is the ATPase at saturating [Ca], n is the Hill coefficient, and pK_Ca_ is the apparent Ca dissociation constant.

### Statistical Analysis

Errors are reported as the standard deviation of the mean, except when noted, and statistical significance was determined by Student’s T test, where p < 0.05 was considered significant, or 1-way ANOVA followed by Tukey post hoc analysis.

### Data availability

The datasets generated during and/or analyzed during the current study are available from the corresponding author on reasonable request.

## Electronic supplementary material

Supplementary Information
