# Supplementary material for: Targeting protein-protein interactions for therapeutic discovery via FRET-based high-throughput screening in living cells
Source: Sci Rep. 2018 Aug 22;8:12560. doi: 10.1038/s41598-018-29685-z (PMC6105598; doi:10.1038/s41598-018-29685-z)
Supplement: Supplementary file 1 — Supplementary Information [file 41598_2018_29685_MOESM1_ESM.docx]

**SUPPLEMENTARY INFORMATION**

**Targeting protein-protein interactions for therapeutic discovery via FRET-based high-throughput screening in living cells**

Daniel R. Stroik^1^, Samantha L. Yuen^1^, Kevyn A. Janicek^1^, Tory M. Schaaf^1^, Ji Li^1^, Delaine K. Ceholski^2^, Roger J. Hajjar^2^, Razvan L. Cornea^1^, and David D. Thomas^1^

*^1^Department of Biochemistry, Molecular Biology, and Biophysics, University of Minnesota, Minneapolis, Minnesota 55455*

*^2^Cardiovascular Research Center, Icahn School of Medicine at Mount Sinai, New York City, New York 10029*

*Corresponding Author

David D. Thomas

Department of Biochemistry,

Molecular Biology and Biophysics

321 Church St. SE, Minneapolis, MN 55455

Telephone: (612) 625-0957

Fax: (612) 624-0632

E-mail: [ddt@umn.edu](mailto:ddt@umn.edu)

**Table S1. Concentration response for SERCA2a-PLB FRET**

| **Compound** | **ΔFLT^a^ (ps)** | **ΔFLT^b^ (ps)** | **EC_50_ FRET (µM)** |
| --- | --- | --- | --- |
| Tenidap | 8 | -68 | ND^c^ |
| Rotenone | 11 | 20 | ND^d^ |
| Candesartan cilexetil | 70 | 181 | 20.1 |
| TBB | 31 | 63 | 73.2 |
| SCH 202676 | 36 | 35 | 1.8 |
| CP 154526 | 48 | 185 | 9.3 |
| FPL 64176 | 19 | 47 | ND^c^ |
| 10058-F4 | 17 | -11 | ND^d^ |
| PF3845 | 30 | 38 | 3.2 |
| Palmitoyl-DL-carnitine chloride | 50 | 189 | 24.9 |
| Ivermectin | 53 | 56 | 5.9 |
| PD-407824 | -103 | -186 | 8.5 |
| K114 | -16 | -102 | 41.3 |
| CP-135807 | -70 | -390 | ND^c^ |
| Retinoic Acid | 27 | -31 | 65.5 |
| Olprinone | 36 | 254 | ND^c^ |
| X80 | -40 | -865 | ND^c^ |
| Ro 41-0960 | -26 | -152 | 22.5 |
| FG7142 | 4 | 10 | ND^d^ |
| AMG9810 | 6 | -1 | ND^d^ |
| Calmidazolium | 45 | -192 | 26.3 |

^a^ Following compound incubation for 20 min at a final concentration of 10 µM, with an uncertainty (1 S.D.) of the measurement of 11 ps.

^b^ Following compound incubation for 20 min at a final concentration of 100 µM, , with an uncertainty (1 S.D.) of the measurement of 11 ps.

^c^ Change in FRET did not reach saturation at 100 µM.

^d^ Change in FRET was below the 3 S.D. threshold of the triplicate screen.

**Table S2. Concentration response for CSR Ca-ATPase Assay**

| **Compound** | **V_max_ @**  **30 µM (i.u.)** | **ΔV_max_ @**  **30 µM (%)** | **EC50 V_max_(µM)** | **pK_Ca_ @30 µM (µM)** |
| --- | --- | --- | --- | --- |
| Tenidap | 0.66 | -1.7 | ND^a^ | 6.09 |
| Rotenone | 0.53 | -22.0 | ND^a^ | 6.07 |
| Candesartan cilexetil | 0.17 | -75.3 | 11.7 | ND^b^ |
| TBB | 0.49 | -27.2 | ND^a^ | 6.07 |
| SCH 202676 | 0.22 | -68.2 | 1.4 | ND^b^ |
| CP 154526 | 0.91 | 34.4 | 1.0 | 6.06 |
| FPL 64176 | 0.52 | -23.0 | ND^a^ | 6.11 |
| 10058-F4 | 0.63 | -7.5 | ND^a^ | 6.08 |
| PF3845 | 0.48 | -28.3 | ND^a^ | 6.11 |
| Palmitoyl-DL-carnitine chloride | 0.22 | -67.1 | 16.1 | ND^b^ |
| Ivermectin | 0.15 | -77.9 | 6.2 | 6.10 |
| PD-407824 | 0.32 | -53.4 | 17.3 | 6.04 |
| K114 | 0.11 | -83.7 | 6.7 | 6.10 |
| CP-135807 | 0.65 | -3.8 | ND^a^ | 6.05 |
| Retinoic Acid | 0.42 | -37.8 | 11.2 | ND^b^ |
| Olprinone | 0.68 | -0.1 | ND^a^ | 6.04 |
| X80 | 0.66 | -2.1 | ND^a^ | 6.09 |
| Ro 41-0960 | 0.53 | -20.9 | ND^a^ | 6.45 |
| FG7142 | 0.67 | -0.7 | ND^a^ | 6.03 |
| AMG9810 | 0.68 | 0.1 | ND^a^ | 6.03 |
| Calmidazolium | 0.08 | -88.7 | 15.9 | ND^b^ |

^a^ Change in V_max_ was <30% of DMSO control

^b^ Did not show Ca-dependent activity due to inhibition.

**Table S3. Concentration response for SSR Ca-ATPase Assay**

| **Compound** | **V_max_ @**  **30 µM (i.u.)** | **ΔV_max_ @**  **30 µM (%)** | **EC50 V_max_(µM)** | **pK_Ca_ @30 µM (µM)** |
| --- | --- | --- | --- | --- |
| Tenidap | 2.66 | 1.8 | ND^a^ | 6.52 |
| Rotenone | 2.64 | 0.7 | ND^a^ | 6.50 |
| Candesartan cilexetil | 0.41 | -84.5 | 7.42 | 6.45 |
| TBB | 2.66 | 1.6 | ND^a^ | 6.50 |
| SCH 202676 | 0.07 | -97.3 | 0.90 | ND^b^ |
| CP 154526 | 3.40 | 35.0 | 2.47 | 6.49 |
| FPL 64176 | 2.64 | 0.7 | ND^a^ | 6.48 |
| 10058-F4 | 2.63 | 0.5 | ND^a^ | 6.48 |
| PF3845 | 2.61 | -0.2 | ND^a^ | 6.44 |
| Palmitoyl-DL-carnitine chloride | 0.57 | -78.1 | 12.23 | 5.51 |
| Ivermectin | 0.32 | -87.6 | 5.75 | ND^b^ |
| PD-407824 | 1.31 | -50.0 | 18.77 | 6.46 |
| K114 | 0.32 | -87.9 | 3.53 | ND^b^ |
| CP-135807 | 2.67 | 2.1 | ND^a^ | 6.46 |
| Retinoic Acid | 2.34 | -10.6 | ND^a^ | 6.48 |
| Olprinone | 2.70 | 3.0 | ND^a^ | 6.50 |
| X80 | 2.46 | -6.1 | ND^a^ | 6.46 |
| Ro 41-0960 | 1.44 | -45.1 | 9.78 | 6.47 |
| FG7142 | 2.63 | 0.5 | ND^a^ | 6.47 |
| AMG9810 | 2.62 | 0.1 | ND^a^ | 6.46 |
| Calmidazolium | 0.01 | -99.5 | 7.43 | ND^b^ |

^a^ Change in V_max_ was <30% of DMSO control

^b^ Did not show Ca-dependent activity due to inhibition.

**Table S4. Effects of Ca and Tg on SERCA2a-PLB biosensor ANOVA**

| **Samples Tested** | **Significant** | **Adjusted P Value** |
| --- | --- | --- |
| -Ca, -Tg vs. +Ca, -Tg | Yes | <0.005 |
| -Ca, -Tg vs. -Ca, +Tg | No | 0.1839 |
| -Ca, -Tg vs. +Ca, +Tg | No | 0.9865 |
| +Ca, -Tg vs. -Ca, +Tg | Yes | <0.005 |
| +Ca, -Tg vs. +Ca, +Tg | Yes | <0.005 |
| -Ca, +Tg vs. +Ca, +Tg | No | 0.1163 |
|  |  |  |

**Table S5. Dependence of CSR Ca-ATPase activity at pCa 6.4 on the concentration of Ro 41-0960 ANOVA**

| **Ro 41-0960 Concentrations Tested** | **Significant** | **Adjusted P Value** |
| --- | --- | --- |
| 0 vs. 2  0 vs. 4 | Yes  Yes | <0.0001  <0.0001 |
| 0 vs. 8 | Yes | <0.0001 |
| 0 vs. 16 | Yes | <0.0001 |
| 0 vs. 24 | Yes | <0.0001 |
| 0 vs. 32 | Yes | <0.0001 |
| 2 vs. 4 | No | 0.5481 |
| 2 vs. 8 | No | 0.1 |
| 2 vs. 16 | Yes | 0.0013 |
| 2 vs. 24 | Yes | <0.0001 |
| 2 vs. 32 | Yes | 0.0002 |
| 4 vs. 8 | No | 0.8897 |
| 4 vs. 16 | Yes | 0.0342 |
| 4 vs. 24 | Yes | 0.0008 |
| 4 vs. 32 | Yes | 0.0039 |
| 8 vs. 16 | No | 0.2507 |
| 8 vs. 24 | Yes | 0.0065 |
| 8 vs. 32 | Yes | 0.0337 |
| 16 vs. 24 | No | 0.4044 |
| 16 vs. 32 | No | 0.8866 |
| 24 vs. 32 | No | 0.9655 |
|  |  |  |

**Figure S1.** **Model testing to determine the time-resolved fluorescence decay function for GFP donor used in this study and the structural state model.** Analysis was first performed on the data from the donor-only sample *F*_D_(t) (GFP-SERCA2a), which was fit by a multi-exponential function (Eq. 5); two exponentials (n = 2 in Eq. 3) were necessary and sufficient for an optimum fit, as shown by χ^2^ optimization in (a). τ_1_ = 1.38 ns, amplitude_1_ = 251; τ_2_ = 2.59 ns, amplitude_2_ = 2188. Then the donor + acceptor data *F*_D+A_(t) was analyzed according to Eq. 6-12, assuming multiple Gaussian components (Eq. 10); two components (N = 2 in Eq. 10) were necessary and sufficient for an optimum fit, as shown by χ^2^ optimization in (b). Distance distribution of best fit two-structural state model corresponds to R_1_ = 5.6 nm, R_2_ = 9.8 nm, FWHM_1_ = 1.6 nm, FWHM_2_ = 1.9 nm, and X_D_ = 0.47.

**Figure S2.** **Chemical structures of SERCA2a-PLB biosensor hits** (a) activators (b) inhibitors

**Figure S3.** **FRET concentration response for SERCA inhibitors** FRET dependence on (0.01-100 µM) SCH 202676 (circle) and Ivermectin (up-triangle) under conditions similar to those in the primary HTS.

**Figure S4.** **SSR Ca-ATPase activity for SERCA inhibitors** ATPase activity from SSR was measured after 20 min incubation with ivermectin (up to 2 µM), SCH 202676 (up to 64 µM) or DMSO control. (inset) Concentration response for limiting activity at high Ca (V_max_).

**Figure S5.** **Uncropped blot images of SERCA2a-PLB biosensor** Immunoblots of homogenates from untransfected HEK293 cells (lane 1), cells expressing GFP-SERCA2a (lanes 2-3), or cells expressing GFP-SERCA2a and increasing amounts of RFP-PLB (lanes 4-8). Antibodies are anti-SERCA2, anti-RFP, anti-PLB and anti-β-actin. * indicates non-specific band. # indicates degradation products.

**Figure S6.** **Reproducible hits assessed across triplicate screens** FLT hits were reproducible (triplicate) using a 3 S.D. threshold (red line). Compounds shown: Ro 41-0960, SCH 202676, CP 154526, and Ivermectin. Each bar (individual screen) indicates the the difference in FLT value compared to the mean value of the corresponding screen.
